# Using EXAFS data to improve atomistic structural models of glasses

**DOI:** 10.1107/S1600577518002072

**Published:** 2018-05-29

**Authors:** Daniel T. Bowron

**Affiliations:** a Science and Technology Facilities Council, ISIS Neutron and Muon Facility, Rutherford Appleton Laboratory, Chilton, Didcot OX11 0QX, UK

**Keywords:** X-ray diffraction, EXAFS, glass structure, empirical potential structure refinement (EPSR)

## Abstract

Quantitative characterization of the atomic structure of multi-component glasses is a long-standing scientific challenge. This is because in most cases no single experimental technique is capable of completely resolving all aspects of a disordered system’s structure. In this situation, the most practical solution for the materials scientist is to apply multiple experimental probes offering differing degrees of insight into a material’s properties. This powerful and widely adopted approach does, however, transfer the characterization challenge to the task of developing a coherent data analysis framework that can appropriately combine the diverse experimental insight into a single, data-consistent, structural model. Here, taking a terbium metaphosphate glass as an example system, it is illustrated how this can be achieved for X-ray diffraction and extended X-ray absorption fine-structure (EXAFS) spectroscopy data, using an empirical potential structure refinement approach. This methodology is based on performing a Monte Carlo simulation of the structure of a disordered material that is guided to a solution consistent with the provided experimental data, by a series of pairwise perturbation potentials operating on a classical reference potential foundation. For multi-component glasses the incorporation of EXAFS data into the resulting bulk structural models is shown to make a critical contribution that is required to properly account for the increase in local structural order that can develop in the melt-quench process of glass formation.

## Abstract 2

A simple scheme for the coherent incorporation of extended X-ray absorption fine-structure (EXAFS) spectroscopy data into the atomistic structure refinement of diffraction data obtained from melt-quenched glasses is demonstrated. The method is shown to significantly improve the quality and consistency of the finally generated structural models.

## 1. Introduction   

For a glass formed from *N* atomic components, 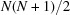 atomic pair correlation functions are required to properly account for the material’s microscopic structure as measured by X-ray, or neutron, diffraction. Although often considered the primary probe of atomic structure, this characteristic of diffraction experiments poses a significant challenge for the disordered materials scientist, as a single measurement will only provide insight into a material’s average structure through a weighted sum of its constituent atomic pair correlations. This limitation makes it difficult to extract from the data an understanding of how the often overlapping atom pair correlations in a disordered material relate to its physical and chemical properties. Over the years, techniques have been developed that allow diffraction experiments to deliver a degree of chemically specific insight into a material’s partial pair correlation functions, for example using anomalous X-ray scattering (Fuoss *et al.*, 1981 ▸) or neutron diffraction with isotopic substitution (Enderby *et al.*, 1966 ▸). However, both of these approaches are generally very difficult, or impossible, to apply to non-ideal or technically unsuitable samples. In contrast, the versatile and widely applicable technique of extended X-ray absorption fine-structure (EXAFS) spectroscopy (Sayers *et al.*, 1971 ▸) is intrinsically chemically specific and provides detailed insight into the short-range atom pair correlations around selected photoabsorbing atom sites within a material. This spectroscopy provides comparable, though shorter-range, structural information to diffraction experiments, and thus has long been seen as an ideal complementary materials characterization probe. In the area of glass science, EXAFS has been a particularly powerful probe and the technique has delivered many significant insights that are now well documented in the literature (*e.g.* Greaves, 1985 ▸; Calas *et al.*, 2014 ▸). From the perspective of a scientist interested in the atomic structure of a disordered material, it is thus highly desirable to be able to coherently combine the bulk-average structural sensitivity of diffraction measurements with the chemically selective capabilities of EXAFS, to arrive at a comprehensive, experimentally consistent, model of a material’s microstructure.

One technique that has been developed to address this challenge is empirical potential structure refinement (EPSR), that was originally conceived by Soper to model the structure of fluids in a manner consistent with experimental diffraction data (Soper, 1996 ▸). This methodology has also been applied to generate data-consistent models of glass systems (*e.g.* Bowron, 2008 ▸; Soper, 2010 ▸; Bouty, 2014 ▸). This methodology was subsequently extended to incorporate guidance from EXAFS data into the ultimately refined models by Bowron & Diaz-Moreno (2007 ▸), and has been extensively used to generate improved models of aqueous salt solutions (Bowron & Diaz-Moreno, 2014 ▸). The method is based upon a classical, canonical (NVT), Monte Carlo simulation of a fluid or structurally disordered solid, based on a set of interatomic reference potentials. These potentials, typically Lennard-Jones type plus Coulomb charges, combined with the fixed atomic density of the model, define an initial atomic configuration that is used to calculate an estimate of a disordered material’s total structure factor as would be measured in a diffraction experiment. This estimated structure factor is then compared with the experimentally measured function, and a set of perturbation potentials derived (Soper, 2005 ▸). These perturbation potentials are then added to the reference potentials and the simulation of the system’s structure is continued. After repeating the simulate, calculate, compare and perturb process over a number of cycles, the configuration of atomic positions within the model is ultimately driven to deliver agreement between the model and experimentally derived scattering functions. Once agreement is reached, the simulation is continued and ensemble-average structural information is extracted.

It is worth noting that the form of the empirical potentials that are developed in the course of the structure refinement process is typically complex and generally does not simply improve just the attractive or repulsive aspects of the global reference potential. Examples of refined empirical potentials can be found in the literature (*e.g.* Mancinelli *et al.*, 2007 ▸).

Based on current conveniently available computational capabilities, *i.e.* personal computer workstations, models produced by this method will typically contain between a few thousand and a hundred thousand atoms, depending on the complexity of the system being investigated. Herein, we will describe how this method can be used to build atomistic models of network glasses that are consistent with diffraction and EXAFS data. Due to subtle differences in the nature of the structural disorder manifest in a glass, produced as a rapidly frozen liquid, to that present in an equilibrium fluid, we will show how EXAFS data can be used to enhance the suitability of Lennard-Jones fluid-optimized potential functions to better describe these systems. As the exemplar system, models will be constructed against X-ray diffraction and EXAFS data collected on a (Tb_2_O_3_)_0.26_(P_2_O_5_)_0.74_ metaphosphate glass (Bowron *et al.*, 1995 ▸).

## 2. Method and results   

As outlined in the *Introduction* [Sec sec1], atomistic models of (Tb_2_O_3_)_0.26_(P_2_O_5_)_0.74_ metaphosphate glass will be constructed using the EPSR technique and refined for consistency against X-ray diffraction data. Two models will be prepared, the first taking no input from the information contained within the available EXAFS data, the second optimized to be consistent with both sets of experimental data.

The structure refinement procedure begins by constructing a cubic simulation box containing 526 Tb atoms, 1474 P atoms and 4474 O atoms, at an atomic density of 0.0695 atoms Å^−3^. Before the addition of any empirical potential contributions derived from the experimental data, these atoms interact using Lennard-Jones and Coulomb forces as shown by equation (1), and parameterized as given in Table 1 ▸,
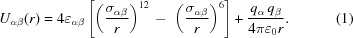
Here, ∊ is the energy that defines the depth of the potential energy well governing the interactions between atoms of type α and β, σ defines the onset (*r* ≃ σ) of the hard-sphere repulsion for each atom, and *q* is the electrical charge assigned to the interacting atoms types. Parameters for interactions between atoms of different types are generated using the standard Lorentz–Berthelot mixing rules σ_αβ_ = (σ_α_ + σ_β_)/2 and ∊_αβ_ = (∊_α_∊_β_)^1/2^. The choice of reference potentials used to initiate the structural models is entirely arbitrary, as they are only required to put the initial structural configuration in a physically and chemically reasonable state. Ideally the reference potential scheme captures: (*a*) the sizes of the atoms in the materials and (*b*) the relative charge stoichiometry expected of the local atomic environments. These reference potentials should also not be too large in energetic magnitude to prevent efficient model evolution in the Monte Carlo modelling engine. The Lennard-Jones potential form allows for easy parameterization of atomic interactions in multi-component glasses and an example of how this can be done has been outlined by Bernasconi *et al.* (2012 ▸). Example parameters for EPSR refinement of a range of oxide glasses can be found in the literature (Bowron, 2014 ▸).

### 2.1. Baseline model of (Tb_2_O_3_)_0.26_(P_2_O_5_)_0.74_ glass   

**Figure 1:**
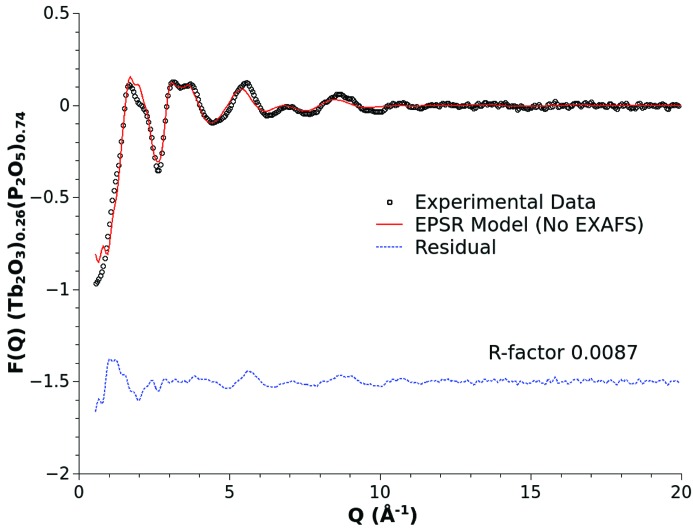
X-ray diffraction data for a (Tb_2_O_3_)_0.26_(P_2_O_5_)_0.74_ metaphosphate glass (open circles), and EPSR fit (solid line) and fit residual (broken line) obtained using the Lennard-Jones + Coulomb charge reference potential model parameterized as shown in Table 1 ▸.

Without reference to the structural information contained in the available EXAFS data, Fig. 1 ▸ shows the result of the EPSR procedure, simply refining the reference potential model (Table 1 ▸) against the supplied X-ray diffraction data. Although not perfect, the EPSR process delivers a reasonable fit to the diffraction data with a computed *R*-factor of ∼0.009 and we will consider this the baseline model for the terbium metaphosphate glass structure. The sensitivity of the model to the various atom pair correlations is related to the scattering weights defined through the well known X-ray interference differential scattering cross section, 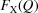, 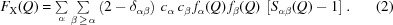
In this equation, *c*_α_ and *c*_β_ are the concentrations of atoms of type α and β, whilst *f*_α_(*Q*) and *f*_β_(*Q*) are their X-ray scattering form factors. *S*_αβ_(*Q*) is the partial structure factor encoding the pair correlations between the atoms, and δ_αβ_ is the Kronecker delta function to avoid double counting the like atom pair terms.

For the (Tb_2_O_3_)_0.26_(P_2_O_5_)_0.74_ glass, the relative percentage weights evaluated at 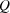 = 0 of the atom pair correlations that constrain to the EPSR model are given in Table 2 ▸.

It is important to note that the overall agreement of the model structure factor with the experimental data is a complex issue. The experimental X-ray diffraction data are not perfect. There are inevitably systematic errors associated with experimental factors such as beam polarization, Compton scattering, attenuation, multiple scattering, sample fluorescence and powder sample packing. Although these have been estimated and corrected to the best of our ability, there will remain some discrepancies which generally contribute low-frequency backgrounds. Generally these residual errors are found to have minimal impact on derived structural conclusions over the interatomic length scales of primary interest, but these residual errors can affect the human appraisal of the quality of a model fit in *Q*-space. The EPSR procedure was specifically designed to minimize the impact of these imperfections on the generated models. Each model is constructed to be consistent with our best understanding of both the data and the fundamental physical properties of the system: the atomic density, the chemical composition, the relative atom sizes and the accepted elemental charges.

The resulting model can now be interrogated for structural information and compared with the original conclusions obtained by direct analysis of the diffraction and EXAFS data (Bowron *et al.*, 1995 ▸). In the direct analysis, the structure of the glass was concluded to be formed from a network of PO_4_ tetrahedra linked by bridging oxygen atoms, where the P—O bond length was 1.58 (5) Å, and within this the terbium atoms were found to be accommodated in predominantly oxygen coordinated sites. On average each terbium atom was found to be coordinated to 7.0 ± 1 oxygen atoms, at an average Tb—O bond distance of 2.25 (5) Å.

**Figure 2:**
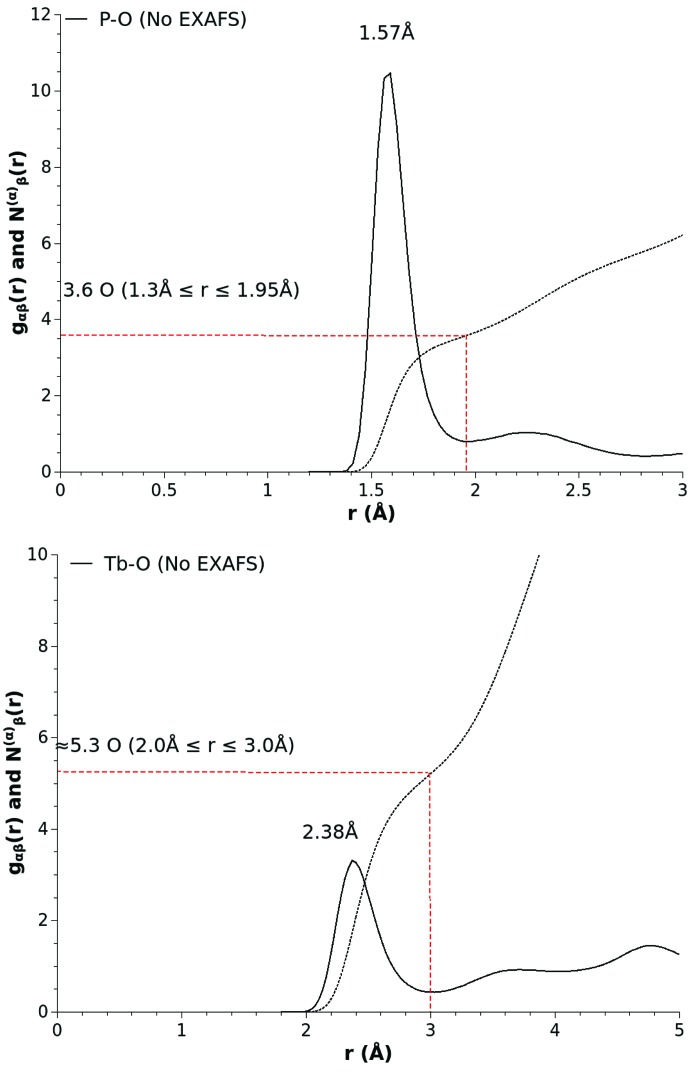
P—O (top panel) and Tb—O (bottom panel) partial pair distribution functions and running coordination numbers extracted from the baseline EPSR model of (Tb_2_O_3_)_0.26_(P_2_O_5_)_0.74_ glass.

Fig. 2 ▸ shows the results obtained from the EPSR model of the diffraction data that relate to the original findings. The refined model presents a phosphate network structure in which each phosphorus atom is surrounded by 3.6 ± 0.1 oxygen atoms at a P—O bond length of 1.57 (5) Å, and a picture of the rare earth atom sites in which each terbium atom is surrounded by 5.3 ± 0.1 oxygen atoms at a distance of 2.38 (5) Å. Clearly, although there are similarities between the original findings for the glass structure, the atomistic model generated by EPSR is highlighting some significant discrepancies, in particular for the rare earth atom sites. On the positive side, the atomistic model does allow us to access all the partial pair distribution functions required to characterize the glass, and these are known to be fully consistent with the primary constraint of the material’s atomic density and estimate of the atomic interactions as encoded by the Lennard-Jones and Coulomb charge parameters. However, as this model was constructed using input only taken from the X-ray diffraction data, *i.e.* without any regard for the information contained in the available EXAFS data, it is worth investigating whether the baseline model is truly consistent with both sets of experimental constraints.

**Figure 3:**
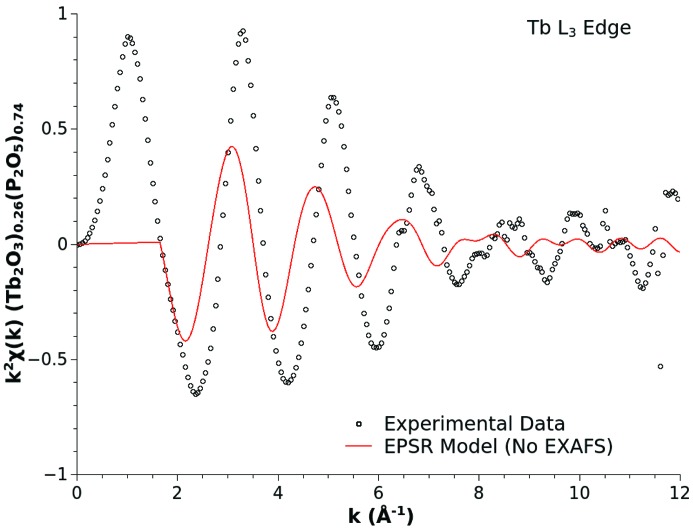
Tb *L*_3_-edge EXAFS data for a (Tb_2_O_3_)_0.26_(P_2_O_5_)_0.74_ metaphosphate glass (black open circles), and theoretical signal (red solid line) calculated from the atomic configurations extracted from the baseline model of the glass.

Fig. 3 ▸ shows the Tb *L*_3_-edge EXAFS signal calculated from the baseline model of the glass; this signal was calculated using the methodology developed by Bowron & Diaz-Moreno (2007 ▸): terbium-atom-centred atomic clusters were extracted from the atomistic model, theoretical EXAFS signals for each cluster were calculated using the *FEFF8* code (Ankudinov *et al.*, 1998 ▸), and then the results from hundreds of sites were ensemble-averaged to give the final result. The *FEFF8* code was used to theoretically estimate the 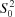 amplitude reduction factor for the signal (= 0.95), and the remaining free parameter in the EXAFS model, 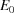, was chosen to deliver the best alignment between the theoretical and experimental signals in the low-*k* range (= 10 eV).

Clearly, the baseline model of the glass does not simultaneously satisfy both the X-ray diffraction and EXAFS data, and so cannot be considered a robust representation of the system.

### 2.2. EXAFS optimized glass model   

Comparing the result of the EXAFS signal calculation from the baseline glass with the experimental signal allows us to conclude that the accommodation of the terbium sites within the model of the phosphate glass matrix is incorrect. The smaller amplitude of the theoretically computed signal tells us that the rare earth atom appears to have too few oxygen near-neighbours, the relatively enhanced signal decay tells us that the baseline model has too much local disorder in the near-neighbour environment, and the relative frequency difference suggests that the Tb—O bond length is a little too long. These are conclusions that equally could be derived from the original direct analysis of the data (Bowron *et al.*, 1995 ▸).

Noting that in this use the EPSR technique is attempting to model the atomic interactions in a glass using small perturbations applied to classical potentials that were originally designed to model equilibrium fluids (Lennard-Jones, 1937 ▸), this is perhaps not a surprise. The modern concept of the *kinetic theory of glass formation*, see for example Shelby (2005 ▸), tells us that, to a first approximation, glass formation from a melt is essentially a process of bypassed crystallization, in which the nucleation and growth of the crystal phase as the liquid solidifies is thwarted by the increase in viscosity of the fluid as it is rapidly quenched. Within this model, the local structure of the nucleating sites within the glass would be expected to be slightly more ordered than when found in the equilibrium fluid prior to quenching.

This hypothesis consequently suggests a means by which we can improve the performance of the EPSR method for modelling the structure of metastable disordered solids, such as glasses. In essence, modifications to the reference potential terms underpinning the model can be made to enhance the degree of local order in specific atom pair correlations. Given the availablility of terbium-centred EXAFS data for this glass, here we will explore the effect of enhancing the short-range Tb—O correlations.

**Figure 4:**
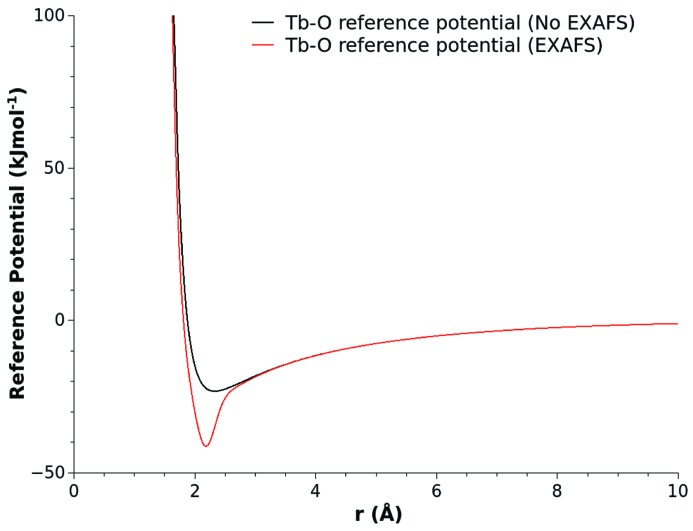
Comparison of the Tb—O Lennard-Jones reference potential used to construct the baseline glass model (black solid line) with the local-order enhanced reference potential (red solid line) used in the EXAFS optimized structure refinement.

**Figure 5:**
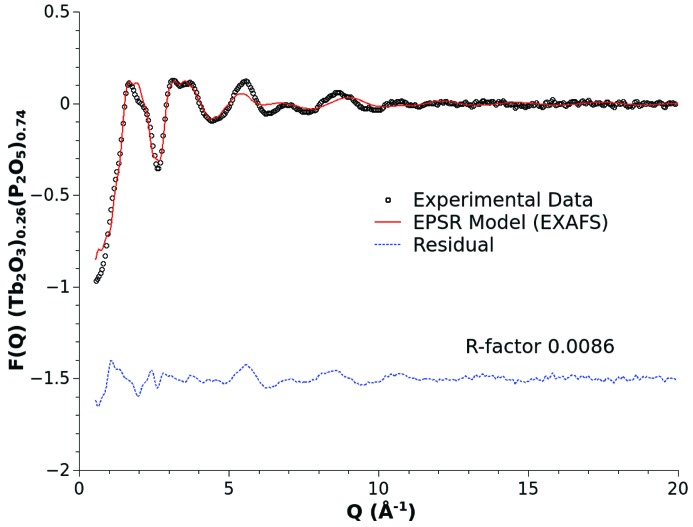
X-ray diffraction data for a (Tb_2_O_3_)_0.26_(P_2_O_5_)_0.74_ metaphosphate glass (black open circles), and EPSR fit (red solid line) and fit residual (blue broken line) obtained using the EXAFS optimized reference potential scheme.

**Figure 6:**
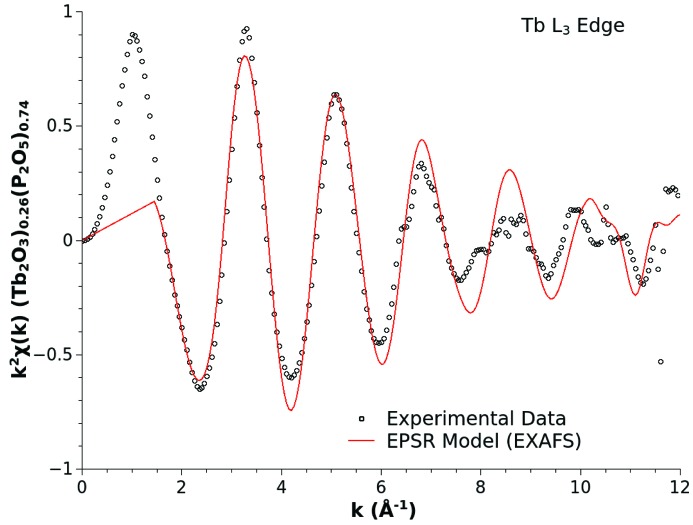
Tb *L*_3_-edge EXAFS data for a (Tb_2_O_3_)_0.26_(P_2_O_5_)_0.74_ metaphosphate glass (black open circles), and theoretical signal (red solid line) calculated from the atomic configurations extracted from the optmized model of the glass in which the local order of the Tb—O interactions has been enhanced.

To enhance the local order in the Tb—O correlations, the Lennard-Jones potential for this interaction has been modified through the addition to the potential defined in equation (1) of a Gaussian trough characterized by a selected position (*P*), width (*W*) and depth (*H*) as per equation (3),
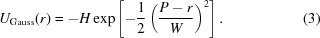
The choice of the Gaussian peak shape is simple to incorporate and allows control of the distance range over which the perturbation is applied. For the purposes of this example, the ultimately selected values of *P*, *W* and *H* were 2.18 Å, 0.15 Å and 15.0 kJ mol^−1^, and the result of this pair potential modification is shown in Fig. 4 ▸. Fig. 5 ▸ shows the resulting fit of the EPSR model refined to the X-ray diffraction data using the local-order enhanced reference potential for the Tb—O interactions. The use of the modified potential has had a negligible effect on the ultimate quality of the fit. Within statistical variations an identical *R*-factor of ∼0.009 has been achieved. Fig. 6 ▸ shows the improvement in the agreement between the model Tb *L*_3_-edge EXAFS signal and the experimental data that the optimized interaction potential delivers. As with the baseline EXAFS calculation, *FEFF8* was used to theoretically estimate the 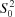 amplitude reduction factor for the signal (= 0.95), and the remaining free parameter in the EXAFS model, 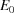, was chosen to deliver the best alignment between the theoretical and experimental signals in the low-*k* range (= 8 eV).

At this stage we now have an atomistic model of the sample that is to first approximation consistent with both the X-ray diffraction data and the EXAFS data relating to the structural accommodation of the terbium atoms in the glass matrix.

## 3. Discussion   

### 3.1. The effect of incorporating EXAFS information into the refined structural model   

**Figure 7:**
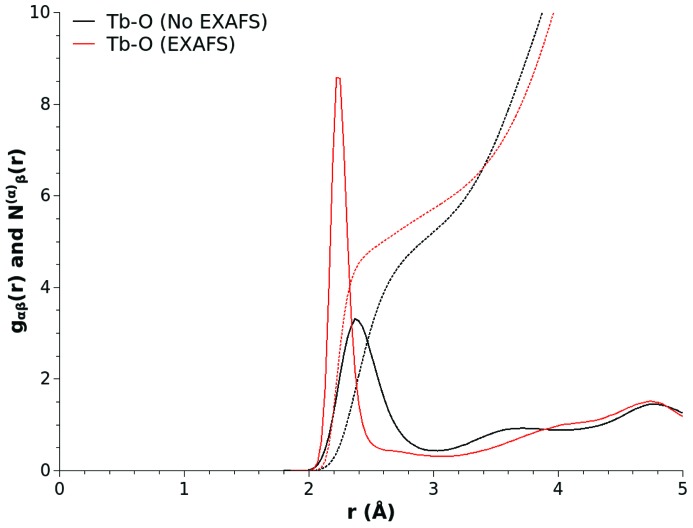
Tb—O partial pair distribution functions and running coordination numbers extracted from the baseline (black solid and black broken lines) and EXAFS optimized (red solid and red broken lines) EPSR models of (Tb_2_O_3_)_0.26_(P_2_O_5_)_0.74_ glass.

To explore the effect that enhancing the local structural order in the short-range Tb—O correlations has had upon the overall glass structure, we can now compare selected functions derived from the baseline and EXAFS optimized models. Fig. 7 ▸ compares the Tb—O pair distribution function and running coordination numbers obtained from each model. The enhancement of the first interaction minimum in the Tb—O reference potential has had a dramatic effect on the localization of the oxygen atoms around the rare earth atoms. The improved refinement has reduced the preferred Tb—O bond length to 2.23 (5) Å whilst the coordination of oxygen atoms around each terbium has increase to ∼6.0 ± 0.1. These revised values are now, within error, in agreement with the originally performed direct analysis of this material.

**Figure 8:**
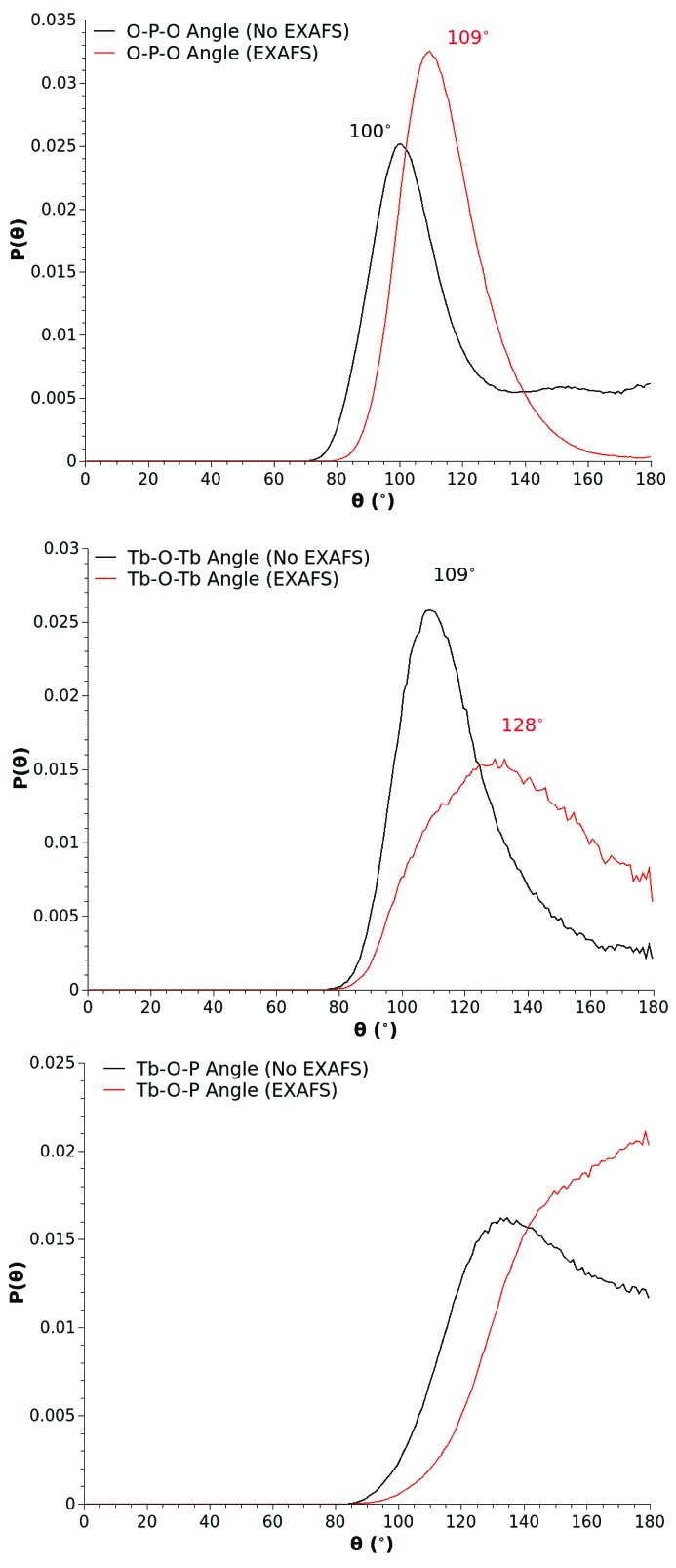
O—P—O, Tb—O—Tb and Tb—O—P bond angle distributions extracted from the baseline (black solid line) and EXAFS optimized (red solid line) EPSR models of (Tb_2_O_3_)_0.26_(P_2_O_5_)_0.74_ glass.

Interestingly, the enhancement of the local order in the terbium environment has had subtle but significant ramifications for the wider connectivity of the glass network. Fig. 8 ▸ shows a selection of bond angle distributions associated with the terbium and phosphorus environments. The EXAFS optimization has had the effect of (i) straightening the Tb—O—P linkages, as seen by a shift in the modal value of the distribution from ∼130° to 180°, (ii) modifying the distribution of terbium sites about linking oxygen atoms away from tetrahedral configurations characterized by a modal value of 109° in the Tb—O—Tb distribution, to higher angles, and (iii) trading the localization of the tetrahedral order in the glass into the O—P—O bond angle distribution where it is most expected, *i.e.* by shifting of the modal value of the O—P—O angle distribution function from 100° to 109°, with a concomittant reduction in the incidence of linear and close to linear O—P—O bond linkages.

### 3.2. The advantages of comprehensive atomistic modelling for glass science   

**Figure 9:**
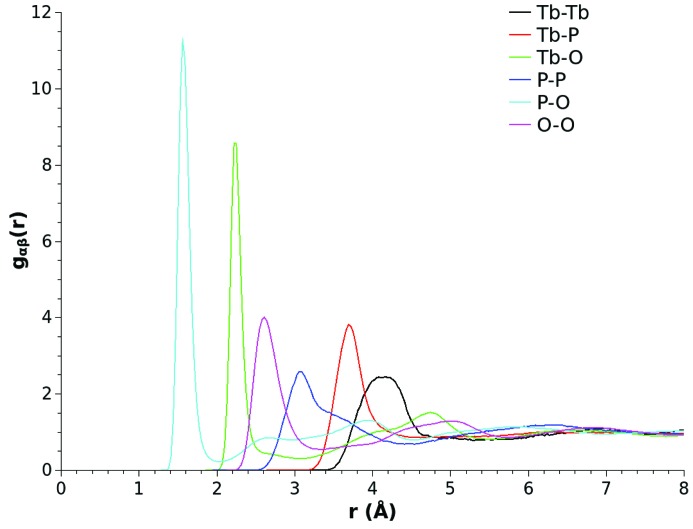
Site–site partial pair distribution functions for a (Tb_2_O_3_)_0.26_(P_2_O_5_)_0.74_ glass derived from an X-ray diffraction and Tb *L*_3_-edge EXAFS optimized EPSR model.

The primary advantage of having an atomistic model of a glass that is consistent with the available experimental data that probes its structure is that it is then possible for the disordered materials scientist to interrogate the atomic configurations for any structural issue of interest. For example, Fig. 9 ▸ shows the complete set of six atomic pair distribution functions that characterize the three-component material that has been studied. One key parameter for the technological application of these materials is the first-neighbour rare earth–rare earth distance, as optical and magnetic properties are closely linked to this value. The optimized model of the investigated glass shows that in this metaphosphate system the first-neighbour Tb—Tb distance is characterized by a relatively broad first correlation peak that is centred at ∼4.1 Å.

**Figure 10:**
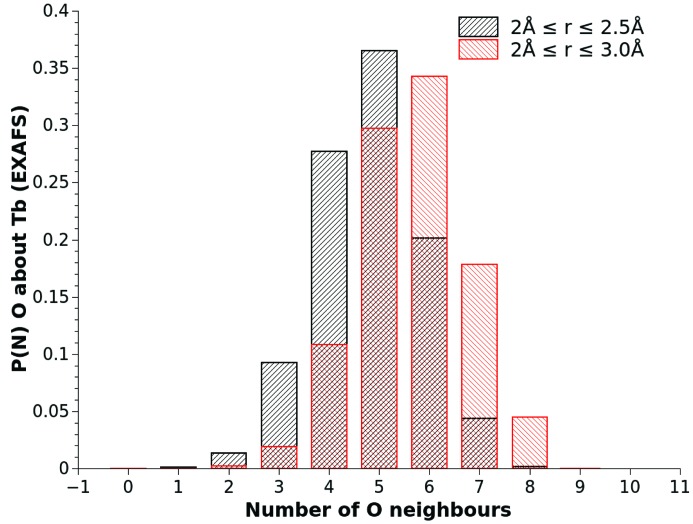
Coordination number probability histograms for oxygen atoms around terbium sites for two different short-range bonding criteria. The black bars show the coordination number distribution for oxygen atoms considered bonded to terbium atoms within a distance criterion of 2.0 Å ≤ *r* ≤ 2.5 Å , and the red bars the distribution if the atoms are considered bonded in the 2.0 Å ≤ *r* ≤ 3.0 Å range.

An additional advantage of having a comprehensive atomic representation of the glass structure is the ability to investigate more than simple average values. This has also been seen in the bond angle information shown in Fig. 8 ▸ but also, for example, in looking beyond the simple mean coordination number of oxygen atoms found around each terbium site. By calculating coordination number histograms from the model structure, we can investigate the distance dependence of the rare earth atom coordination. This allows us to obtain an understanding of how diverse the distribution of sites within the material is (see Fig. 10 ▸). In this case, the distribution functions tell us that the modal value of oxygen coordination is five or six neighbours, depending on how tightly the bonding criterion is defined, but that there are also a significant fraction of low- (three, four) or high- (seven) oxygen coordination sites within the glass network structure.

## 4. Conclusions   

Formally a three-component glass, such as the system under current investigation, requires six partial structure factors and therefore six complementary scattering experiments to fully characterize the pairwise interactions between its constituent atoms. In this study only one X-ray diffraction experiment was performed to drive an atomistic modelling process, and this single data set only provides a primary constraint on the ultimate structural solution that is formed from a weighted sum of the partial structure functions. To get round this limitation the technique of empirical potential structure refinement (Soper, 1996 ▸) has been used, as the method provides the ‘missing’ information required to solve the structure, using estimations derived from a reference potential based atomistic model that forms the starting point for the structure refinement process. This work has subsequently demonstrated that although this process can be performed, the final result is not necessarily sufficient to properly constrain many subtle details of the system’s structure. For materials systems such as atomic network glasses where the structure refinement process is not assisted by knowledge of molecular geometry, the most fruitful application of EPSR and similar methods will require additional structural information and insight to be provided from complementary techniques such as EXAFS spectroscopy.

This study has consequently highlighted how EXAFS data can be used to enhance the reliability and utility of atomistic models of glasses derived from structure refinement of X-ray (and neutron) diffraction data. Using the EPSR technique developed by Soper (1996 ▸), this work has illustrated how improvement of local structure features in the model, observed by EXAFS spectroscopy, could be achieved through a small modification made to a first-neighbour interaction minimum, within an underpinning classical reference potential scheme. Beyond this, the methodology of using EXAFS information to enhance structural models of glasses is in principle extensible to other model building methods used within the field. Potential targets include conventional Monte Carlo or molecular dynamics simulation approaches, or comparable methods to EPSR such as the widely used reverse Monte Carlo technique (McGreevy, 2001 ▸), that is also being developed to make use of classical potential schemes (Gereben & Pusztai, 2012 ▸).

Finally, for readers interested in trying empirical potential structure refinement for themselves, source code, supporting documentation and executable versions of the program can be freely obtained by download from the Science and Technology Facilities Council (EPSR, 2017 ▸).

## Figures and Tables

**Table 1:** Lennard-Jones and charge parameters used for the (Tb_2_O_3_)_0.26_(P_2_O_5_)_0.74_ metaphosphate glass reference potentials For modelling network glass structures it is computationally advantageous to reduce the magnitude of the formal charges used to parameterize the electrostatic interactions. These charges provide a strong coupling to the local chemical stoichiometry of the atoms within the system but full formal charges would hinder the efficiency of the EPSR Monte Carlo modelling engine to explore the space of structural configurations as the empirical potential develops to drive the structure refinement process.

Atom	∊ (kJ mol^−1^)	σ (Å)	*q* (e)
Tb	0.8	1.85	0.3
P	0.8	0.62	0.5
O	0.65	0.27	−0.2

**Table 2:** Relative percentage weights of the contribution of the atom pair correlations to the X-ray total structure factor of a (Tb_2_O_3_)_0.26_(P_2_O_5_)_0.74_ glass The values are calculated for the *Q* = 0 limit of the atomic form factors.

Pair correlation	X-ray weight (%)
Tb—Tb	13.8
Tb—P	17.9
Tb—O	28.9
P—P	5.8
P—O	18.7
O—O	15.1
